# Topoisomerase I in Human Disease Pathogenesis and Treatments

**DOI:** 10.1016/j.gpb.2016.02.004

**Published:** 2016-05-12

**Authors:** Min Li, Yilun Liu

**Affiliations:** Department of Cancer Genetics and Epigenetics, Beckman Research Institute, City of Hope, Duarte, CA 91010-3000, USA

**Keywords:** Topoisomerase 1, Cancer, Autism, Scleroderma, DNA replication, Transcription

## Abstract

Mammalian **topoisomerase 1** (TOP1) is an essential enzyme for normal development. TOP1 relaxes supercoiled DNA to remove helical constraints that can otherwise hinder **DNA replication** and **transcription** and thus block cell growth. Unfortunately, this exact activity can covalently trap TOP1 on the DNA that could lead to cell death or mutagenesis, a precursor for tumorigenesis. It is therefore important for cells to find a proper balance between the utilization of the TOP1 catalytic activity to maintain DNA topology and the risk of accumulating the toxic DNA damages due to TOP1 trapping that prevents normal cell growth. In an apparent contradiction to the negative attribute of the TOP1 activity to genome stability, the detrimental effect of the TOP1-induced DNA lesions on cell survival has made this enzyme a prime target for **cancer** therapies to kill fast-growing **cancer** cells. In addition, cumulative evidence supports a direct role of TOP1 in promoting transcriptional progression independent of its topoisomerase activity. The involvement of TOP1 in transcriptional regulation has recently become a focus in developing potential new treatments for a subtype of **autism** spectrum disorders. Clearly, the impact of TOP1 on human health is multifold. In this review, we will summarize our current understandings on how TOP1 contributes to human diseases and how its activity is targeted for disease treatments.

## Introduction

**Figure 1:**
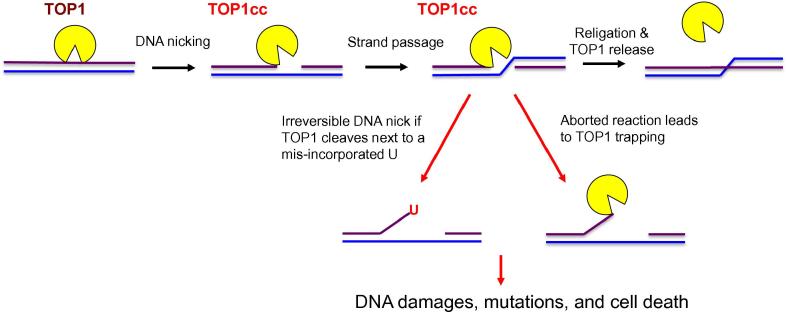
Illustration of TOP1 DNA cleavage reaction The TOP1 DNA cleavage reaction is initiated by the binding and DNA nicking (purple line) of TOP1 to form a TOP1cc complex that covalently links TOP1 to the DNA. The intact DNA strand (blue line) passes through the DNA nick before the nick is religated, followed by the release of TOP1 from the DNA. TOP1 DNA cleavage next to a misincorporated ribonucleotide U or an aborted TOP1cc reaction can lead to mutations and cell death. TOP1 is shown in yellow and the two DNA strands are shown in purple and blue, respectively. TOP1, topoisomerase 1; TOP1cc, TOP1–DNA cleavage complex; U, uridine.

Topoisomerase 1 (TOP1) is a highly conserved enzyme that can be found in both prokaryotes and eukaryotes. In the mammalian system, TOP1 is an essential enzyme for normal development [1]. A major function of TOP1 is to relax supercoiled DNA and alleviate the DNA helical constraints [2], [3]. This is achieved by the binding of TOP1 to the supercoiled DNA, followed by the cleavage of one strand of the duplex DNA to create a nick, allowing the duplex DNA to untwist and relax (Figure 1) [4]. DNA supercoiling is a naturally-occurring biological process when a DNA replisome or an RNA polymerase (RNAP) unwinds and translocates on the DNA to synthesize DNA or RNA. If not removed, these supercoiled DNA can hinder the progression of the replication fork or RNAP. In addition, negatively supercoiled DNA can facilitate the formation of RNA:DNA hybrids, or R-loops, between DNA template and the newly-synthesized RNA. If not resolved, R-loops can stall further transcription and DNA replication forks, leading to DNA double-strand break (DSB) formation [5]. TOP1 is known to interact directly with the active form of RNAPII and localize to transcriptionally-active regions (TARs) of the genome [2], [3]. It has been suggested that TOP1 may aid to suppress R-loop formation by removing supercoiled DNA during RNAPII-dependent transcription [4], [5].

In addition to its function in relaxing supercoiled DNA, cumulative evidence supports a direct role of TOP1 in transcriptional regulation. For example, during transcription, RNAPII pauses at initiation and splice sites [6], while TOP1 has been proposed to hold RNAPII at the promoter-proximal pause site [7]. Nonetheless, the exact molecular mechanism by which TOP1 pauses RNAPII at the initiation site remains to be defined. Furthermore, TOP1 has been shown to promote the recruitment and assembly of spliceosome at TARs [8], [9], [10], and this function may be contributed by a potential TOP1-associated kinase activity to phosphorylate splicing factors [9]. Efficient recruitment and coupling of RNA processing factors to the TARs are critical for ensuring uninterrupted production of full-length mature mRNA. In addition, spliceosome assembly onto nascent RNA transcript has important implications for genome stability as well, because the binding of RNA processing factors to the newly-transcribed RNAs can also prevent these RNA strands from invading the DNA template to generate R-loops [5], [9], [11]. The involvement of TOP1 in spliceosome assembly may explain why TOP1 is important for transcriptional progression and R-loop suppression. Nonetheless, whether TOP1 functions as a protein kinase for the spliceosome assembly remains in great debate, as evidence also suggests that TOP1 is unlikely the only or the primary kinase that phosphorylates splicing factors [12], [13].

The dynamic functions of TOP1 in DNA replication and transcription provide important clues to why TOP1 is essential for development in the mammalian system. However, because TOP1 forms a covalent link intermediate, known as TOP1–DNA cleavage complex (TOP1cc), with the 5′ phosphate group of the DNA during the topoisomerase reaction, the TOP1 activity can generate toxic DNA lesions due to a naturally-aborted topoisomerase reaction, leaving the TOP1 covalently trapped on the DNA (Figure 1) [14]. Alternatively, single-strand breaks (SSBs) accumulate due to irreversible DNA cleavage by TOP1 adjacent to a misincorporated ribonucleotide [15]. The presence of these TOP1cc and DNA lesions may lead to cell death or mutagenesis, a precursor for tumorigenesis. Therefore, the topoisomerase activity of TOP1 is a double-edged sword and can have both positive and negative consequences on genome integrity and normal cell growth.

**Figure 2:**
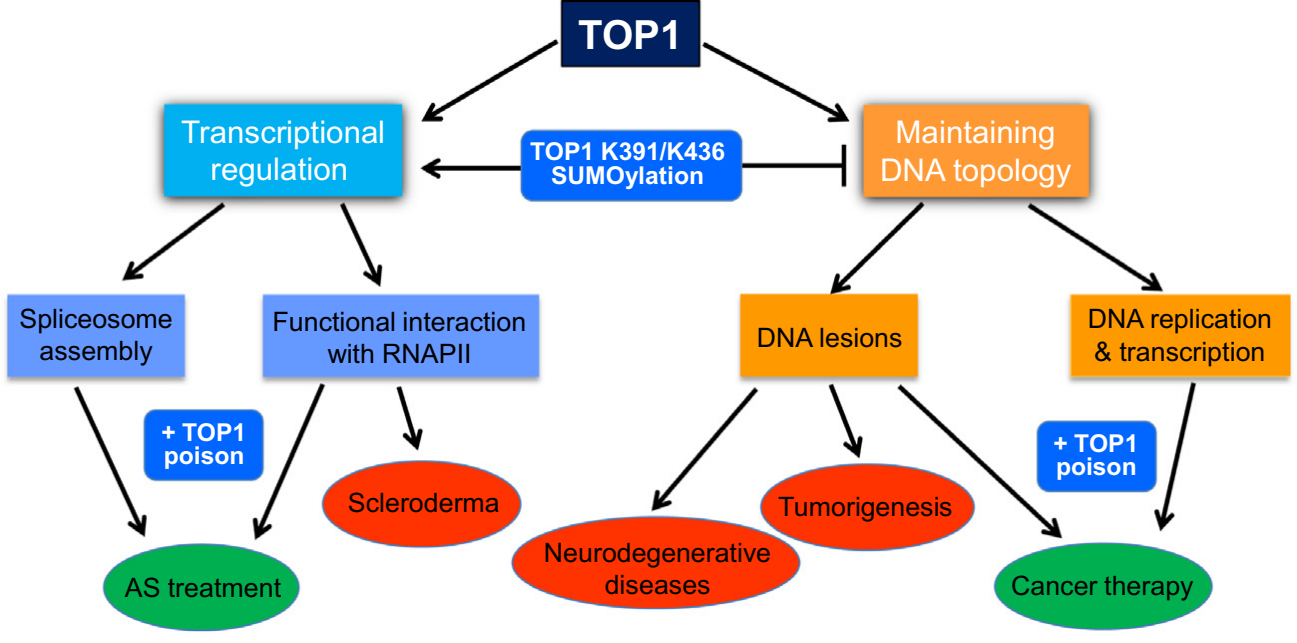
The positive and negative attributes of TOP1 action to human health Summary diagram showing the functions of human TOP1, the regulation of these functions by SUMOylation and its potential link to human diseases (indicated in red) and therapies using TOP1 poisons (indicated in green). TOP1, topoisomerase 1; RNAPII, RNA polymerase II; AS, Angelman syndrome.

In addition, the potential direct involvement of TOP1 in transcriptional regulation [7], [8], [9], [10] suggests that TOP1 dysfunction may alter transcriptional landscape, leading to abnormal cellular functions. It is therefore not surprising that several human diseases have been linked to TOP1 regulation and activity. In this review, we will discuss the human diseases that may be linked to TOP1 and the mechanism by which the TOP1 activity may contribute to the etiologies of these diseases (Figure 2). In addition, we will also overview how the poisonous effect of TOP1cc on cell growth has benefited cancer treatments and how the ability in changing the transcriptional landscape by TOP1 has become a focus for developing possible novel strategy to treat genetic diseases.

## TOP1 in tumorigenesis

In yeast, TARs are prone to mutations that arise as erroneous repair of TOP1cc created by TOP1-mediated removal of supercoiled DNA or irreversible DNA nick generated by the TOP1 cleavage next to a misincorporated ribonucleotide [14], [15]. The mutagenic potential of the TOP1 activity demonstrated in yeast suggests that if the same activity was to exist in humans, TOP1 activity may be a significant contributor to tumorigenesis. However, to date, very little research has been done to evaluate the connection between TOP1 activity and cancer risk. It is possible that in human cells, TOP1 activity is regulated differently at TARs, such that TOP1 in human cells does not produce a high mutation rate during transcription. Indeed, recently, new studies from our laboratory have shown that human cells actively suppress the topoisomerase activity of TOP1 at TARs via novel SUMO modifications at the lysine residues K391 and K436, thereby reducing TOP1-induced DNA damage (Figure 2) [10]. These SUMOylation sites are located within the catalytic core of the enzyme and are only found in mammals but not in yeast. Therefore, our studies suggest that humans have evolved a mechanism to minimize this type of transcription-associated genome instability caused by the TOP1 activity. Nonetheless, the protective effect of TOP1 K391 and K436 SUMOylations against TOP1-induced DNA damage during transcription also strongly points toward the possibility that a SUMOylation defect on these residues could lead to genome instability, mutagenesis, and cancer. This defect could be a consequence of a mutation within the SUMOylation motif sequence for either K391 or K436. Alternatively, mutations that lead to a defect in the interaction between TOP1 and its SUMO conjugation enzymes may also contribute to elevated TOP1 activity at TARs and increase in transcription-induced mutagenesis. Clearly, more studies on the TOP1 mutations that affect these inhibitory SUMOylations are needed to establish a connection between tumor pathogenesis and a dysfunction in the regulation of TOP1 activity in human cells.

## TOP1 in cancer therapy

While the accumulation of TOP1cc on DNA can lead to cell death, paradoxically, it is this toxic effect that makes the TOP1 activity a prime target for cancer therapy since ancient times. Camptothecin (CPT) is a natural herbal compound derived from *Camptotheca* tree native to China and has been used in traditional Chinese medicine for thousands of years due to its anti-tumor activity [16]. It was not until in the 1980 s, TOP1 was identified as the target for CPT [17]. Since then, the synthetic analogs of CPT, such as irinotecan and topotecan, have been developed as chemotherapeutic drugs, which have been approved both in the United States and in Europe for treating several aggressive and metastasized cancers [18]. CPT and its analogs are TOP1 poisons that have high affinity to the DNA-bound TOP1 molecules that are actively catalyzing the removal of supercoiled DNA [16]. The binding of TOP1 poisons to the active TOP1–DNA complex prevents the completion of the topoisomerase reaction and traps TOP1 covalently onto DNA to create DNA damage and induce cell death [18]. In addition, TOP1 poisons were found to sensitize cells to radiation therapy [19], [20], increasing their potential usefulness in cancer therapies.

Nonetheless, while fast-growing cells, such as cancer cells, are more vulnerable to DNA damage-induced cell death, the current dosages of CPT and its analogs used in chemotherapies can induce life-threatening side effects, including hematological toxicities, neutropenia, and diarrhea [21], [22]. Therefore, the development of new strategies to improve the efficacy of TOP1 poisons by increasing the sensitivity of fast-growing cancer cells to these drugs is an active research area. One way to sensitize cells to TOP1 poisons is to prevent the repair and removal of the TOP1 covalent adducts on the DNA. However, this approach may be complicated by the fact that there are several redundant DNA repair pathways that are potentially involved in repairing TOP1-induced DNA damages [23]. Alternatively, since TOP1 poisons are thought to target only those TOP1 molecules that are actively catalyzing the topoisomerase reaction on the DNA, increasing TOP1 activity in cancer cells may enhance their sensitivity to killing by TOP1 poisons. Indeed, it has been observed that patients with higher TOP1 activity level responded to irinotecan- or topotecan-based chemotherapy better than those individuals with lower TOP1 activity level [24], [25]. However, the question is, is it possible to transiently increase TOP1 activity in a cell? Our studies have shown that a defect in TOP1 K391 and K436 SUMOylations increases TOP1 activity [10], thereby causing more TOP1cc on the DNA and sensitizing human cells to the effects of TOP1 poisons. We thus suggest that developing a mechanism to block TOP1 K391 and K436 SUMOylations may be a useful therapeutic strategy to hypersensitize cells to TOP1 poisons during chemotherapy.

## TOP1 in neurodegenerative diseases

The removal of TOP1cc and the repair of TOP1cc-induced DNA SSB lesions require the activation of ATM-dependent DNA damage response, which phosphorylates and activates tyrosyl-DNA phosphodiesterase-1 (TDP1) to remove the covalently-trapped TOP1 from DNA [26]. Mutations in ATM and TDP1 have been linked to neurodegenerative diseases known as ataxia telangiectasia (A-T) and spinocerebellar ataxia with axonal neuropathy (SCAN-1), respectively [27], [28]. Brain functions are significantly impaired in both diseases, and one of the symptoms in these diseases is difficulty in speech, or dysarthria. Because both ATM and TDP1 are important for repairing DNA damages induced by TOP1 poisons [26], a recent study used mouse model to demonstrate that the accumulation of TOP1cc-associated DNA lesions due to defective ATM or TDP1 contributes to the pathogenicity of the neuronal degeneration phenotypes in neural tissue [29]. Interestingly, transient development of dysarthria has been reported in rare cases during TOP1 poison-based chemotherapies due to their neurotoxicity [30]. More studies should be done to understand the genetic backgrounds of those patients, who suffered dysarthria or other neurotoxic side effect during chemotherapeutic treatments using TOP1 poisons, to see if ATM or TDP1 are potential biomarkers for their susceptibility to these symptoms.

## TOP1 in autoimmune diseases

High titer TOP1 autoimmune antibodies are among the most common features of scleroderma [31], [32] and are associated with a poor prognosis and a high mortality rate as well [32], [33]. Scleroderma describes a group of diseases characteristic of hardening of the skin and connective tissues caused by production of autoimmune antibodies. The majority of scleroderma patients produce autoimmune antibodies against their own nuclear constituents, which are not normally accessible to the immune system in healthy individuals [33]. An autoimmune response can be triggered by an abnormally high level of apoptosis or a defect in the clearing of apoptotic cells, both of which can lead to an increase in the presentation of the apoptotic nuclear contents to the immune system [34]. In addition, unusual post-translational modifications may cause the immune system to no longer recognize and tolerate the polypeptide as a self-protein [34], [35]. Indeed, the degree of SUMOylation, such as in the case of TOP1, is significantly elevated in scleroderma tissues [36], [37].

Epitope mapping indicates that α-TOP1 autoantibodies are highly reactive to its catalytic domain [31], [38]. However, the reason for which an individual develops a chronic autoimmune response against TOP1 and the consequence of the binding of these autoimmune antibodies to TOP1 remain unclear. Interestingly, patients with autoimmune antibodies against RNAPII are often positive for α-TOP1 autoantibodies as well [39], but the reason for the high frequency of RNAPII-TOP1 co-autoimmune response is not known either. The contribution of TOP1 to scleroderma is not limited to the production of α-TOP1 autoantibodies. In many scleroderma tissues, there are also a decrease in TOP1 catalytic activity and an increase in TOP1 SUMOylation [37], but the nature of this SUMOylation and the significance of these phenomena to scleroderma pathogenesis are yet to be defined. Since studies from our laboratory revealed that transcription-associated TOP1 K391 and K436 SUMOylation suppresses TOP1 activity while facilitating the TOP1–RNAPII interaction, it would be interesting to determine if TOP1 K391/K436 SUMO modification is deregulated in scleroderma. While a defect in transcription-associated TOP1 K391/K436 SUMOylation could lead to DNA damage and genome instability, hyper K391/K436 SUMOylation would be predicted to enhance the level of TOP1–RNAPII complexes in cells and alter transcriptional landscape, leading to transcriptional stress and increased programed cell death. The elevated level of cells undergoing apoptosis is expected to lead to increased presentation of the TOP1–RNAPII complex to the immune system, resulting in autoimmunity. The increased cell deaths could also contribute to organ failure and fibrosis observed in scleroderma patients.

## TOP1 in autism

Interestingly, in addition to being widely used in cancer therapy, TOP1 poisons were recently shown to alleviate Angelman syndrome, a subtype of autism spectrum disorders (ASD) by suppressing the exceptionally long, antisense RNA transcript *UBE3A-ATS* [40], [41]*. UBE3A-ATS* blocks the expression of its sense gene *UBE3A* that is important for preventing the disease [40], [41]. Nonetheless, how TOP1 poisons affect *UBE3A-ATS* expression remains unclear. Treatment with TOP1 poisons, such as CPT or topotecan, can lead to the reduced expression of many genes in both yeast and human cells [42]. This transcriptional blockade was originally attributed to either the presence of unresolved supercoiled DNA or the accumulation of covalently-trapped TOP1 on the genomic DNA [43]. However, recent observations demonstrated that TOP1 poisons only reduce the expression of exceptionally-long and highly-transcribed genes with median gene length of 66 kb, while up-regulating the expression of shorter genes that are normally expressed at low levels [44]. In addition, similar transcriptional interference can also be achieved by TOP1 depletion [40], suggesting that the effect of TOP1 poisons on transcriptional progression is not due to DNA damage caused by the formation of TOP1cc, which requires the presence of catalytically-active TOP1. In addition, the level of transcriptional suppression by TOP1 poisons not only depends on the gene length but also positively correlates with the number of introns in the gene [44].

Since TOP1 has been implicated in the recruitment and the assembly of spliceosome at TARs to promote efficient transcriptional progression [8], [9], [10], it is possible that TOP1 poisons may influence the spliceosome assembly to exert inhibitory effects on gene expression in an intron-dependent manner [44]. Since spliceosome assembly on the newly-synthesized mRNA also contributes to suppressing R-loops [5], [9], [11], the possible effect of TOP1 poisons on spliceosome assembly is consistent with the observation that topotecan stabilizes R-loop formation, which correlates with the inhibition of the expression of *UBE3A-ATS* [45].

In summary, TOP1 poisons could be useful for the treatment of Angelman syndrome or other genetic disorders that may be suppressed by blocking expression of long genes. However, these compounds are toxic chemotherapeutic drugs and are not safe for long-term use. Therefore, a better understanding of the mechanism by which TOP1 poisons block long gene expression is necessary in aiding researchers to identify novel alternative strategies to target TOP1 in gene expression regulation.

## Competing interests

The authors have declared that no competing interests exist.
